# mTOR Inhibitors Alone and in Combination with JAK2 Inhibitors Effectively Inhibit Cells of Myeloproliferative Neoplasms

**DOI:** 10.1371/journal.pone.0054826

**Published:** 2013-01-31

**Authors:** Costanza Bogani, Niccolò Bartalucci, Serena Martinelli, Lorenzo Tozzi, Paola Guglielmelli, Alberto Bosi, Alessandro M. Vannucchi, Associazione Italiana per la Ricerca sul Cancro AGIMM Gruppo Italiano Malattie Mieloproliferative

**Affiliations:** Department of Medical and Surgical Care, Section of Hematology, University of Florence, Florence, Italy; West Virginia University School of Medicine, United States of America

## Abstract

**Background:**

Dysregulated signaling of the JAK/STAT pathway is a common feature of chronic myeloproliferative neoplasms (MPN), usually associated with *JAK2*V617F mutation. Recent clinical trials with JAK2 inhibitors showed significant improvements in splenomegaly and constitutional symptoms in patients with myelofibrosis but meaningful molecular responses were not documented. Accordingly, there remains a need for exploring new treatment strategies of MPN. A potential additional target for treatment is represented by the PI3K/AKT/mammalian target of rapamycin (mTOR) pathway that has been found constitutively activated in MPN cells; proof-of-evidence of efficacy of the mTOR inhibitor RAD001 has been obtained recently in a Phase I/II trial in patients with myelofibrosis. The aim of the study was to characterize the effects *in vitro* of mTOR inhibitors, used alone and in combination with JAK2 inhibitors, against MPN cells.

**Findings:**

Mouse and human *JAK2*V617F mutated cell lines and primary hematopoietic progenitors from MPN patients were challenged with an allosteric (RAD001) and an ATP-competitive (PP242) mTOR inhibitor and two JAK2 inhibitors (AZD1480 and ruxolitinib). mTOR inhibitors effectively reduced proliferation and colony formation of cell lines through a slowed cell division mediated by changes in cell cycle transition to the S-phase. mTOR inhibitors also impaired the proliferation and prevented colony formation from MPN hematopoietic progenitors at doses significantly lower than healthy controls. JAK2 inhibitors produced similar antiproliferative effects in MPN cell lines and primary cells but were more potent inducers of apoptosis, as also supported by differential effects on cyclinD1, PIM1 and BcLxL expression levels. Co-treatment of mTOR inhibitor with JAK2 inhibitor resulted in synergistic activity against the proliferation of *JAK2*V617F mutated cell lines and significantly reduced erythropoietin-independent colony growth in patients with polycythemia vera.

**Conclusions/Significance:**

These findings support mTOR inhibitors as novel potential drugs for the treatment of MPN and advocate for clinical trials exploiting the combination of mTOR and JAK2 inhibitor.

## Introduction

The chronic myeloproliferative neoplasms (MPN), which include polycythemia vera (PV), essential thrombocythemia (ET) and primary myelofibrosis (PMF) [1], are characterized by a V617F point mutation in the exon 14 of Janus Kinase 2 (*JAK2)* that occurs in 95% of PV and 60% of ET or PMF patients [2], [3]. In addition, *JAK2* exon 12 mutations are detected in 2–3% of PV [4] and mutations in *MPL*, the receptor for thrombopoietin, have been reported in 5–10% of ET and PMF patients [5]–[8]. These molecular abnormalities result in a constitutive activation of the JAK/signal transducer and activator of transcription (STAT) signaling pathway and contribute to cytokine hypersensitivity and cytokine independent growth of the mutant cells, as exemplified by the erythropoietin-independent erythroid colonies (EEC) typically found in most PV patients [9], [10]. Transplant, transgenic and conditional knock-in murine models have shown that expression of mutated *JAK2*V617F is sufficient to recapitulate a MPN phenotype [11]–[19], indicating that constitutive activation of the JAK2/STAT pathway deserves a central role in the pathogenesis of MPNs.

Therefore, activated JAK2 has become an attractive target for therapy. In preclinical models, ATP-competitive JAK2 inhibitors prevented the proliferation of *JAK2*V617F mutant cells *in vitro* and mitigated myeloproliferation in *JAK2*V617F transgenic animals [20], [21]. When employed in clinical trials, JAK2 inhibitors produced significant clinical improvement in patients with myelofibrosis [22], [23] or hydroxyurea-resistant PV or ET [24] at such an extent that the JAK1 and JAK2 inhibitor ruxolitinib has been approved recently by FDA for the treatment of patients with intermediate and high risk myelofibrosis based on the results of two large phase III trials [25], [26] demonstrating rapid and sustained reduction of splenomegaly and improvement of constitutional symptoms. However, only modest reduction of the *JAK2*V617F burden were documented [23]. This is consistent with the observation that the disease-initiating cell population in *JAK2*V617F knock-in mice was not affected by treatment with the JAK2 inhibitor TG101348 [12], supporting current notion that *JAK2* mutation presumably does not represent the driver mutation in MPN [2], [27]. Thus, it seems unlikely that eradication of the MPN clone can be achieved with (available) JAK2 inhibitors; therefore, novel drugs and more effective therapeutic strategies need to be sought. In this regard, it has been shown that co-treatment of the HDACi panobinostat and the JAK2 inhibitor TG101209 resulted in greater attenuation of JAK/STAT signaling in human and mouse *JAK2*V617F-mutated cells and increased cytotoxicity against MPN CD34^+^ cells compared to individual drugs [28].

Enhanced activation of downstream pathways other than JAK/STAT, including the phosphatidylinositol 3-kinase (PI3K) and extracellular signal-regulated kinase (ERK) pathways, has been documented in *JAK2*V617F mutated cells [29], [30]. The serine/threonine protein kinase B/Akt is downstream of PI3K [31]; it is a key regulator of many cellular processes including cell survival, proliferation and differentiation, and has been found dysregulated in many cancer cells [32]. The main target of activated Akt is the serine/threonine kinase mTOR that exists in two complexes, TORC1 and TORC2. TORC1, formed with raptor, controls the level of cap-dependent mRNA translation and phosphorylates effectors such as the eukaryotic initiation factor 4E-binding protein 1 (4E-BP1) and S6 kinase 1 (S6K1); it is strongly inhibited by rapamycin and its derivatives. In turn, phosphorylated 4E-BP1 affects the translation activation of several genes, including cyclin D1, Bcl-2, Bcl-X_L_ and VEGF, whereas S6K1 regulates cell growth by phosphorylating key targets such as eukaryotic initiation factor 4E (eIF4E), mTOR itself and elongation-2 kinase. Both eIF4E and S6K1 have been involved in cellular transformation and are overexpressed in some poor-prognosis cancers [32], [33]. Additional components of mTORC1 include mammalian LST8/G-protein β-subunit like protein (mLST8/GβL) and the recently identified partners PRAS40 and DEPTOR [34], [35]. mTOR also combines with Rictor in mTORC2, that is largely rapamicin insensitive, and is composed of GβL and mammalian stress-activated protein kinase interacting protein 1 (mSIN1). mTORC 2 is also involved in the phosphorylation of Akt at Ser473, that might, in some instances, mediate a negative feedback loop to dampen IRS-1/PI3K/AKT signalling. To overcome possible limitations and drawbacks of allosteric mTOR inhibitors, such as rapamycin and RAD001, novel molecules acting as competitive inhibitors of the mTOR ATP active site have been developed; one of these, PP242 strongly suppresses both mTORC1 and mTORC2-mediated activities [36] and exerted potent cytotoxicity against leukemia cells [37].

Although Akt was found constitutively activate in *JAK2*V617F mutated cells *in vitro* [29], [30] and in V617F transgenic [38] or knock-in mice [19], the contribution of PI3K/Akt signaling to the pathogenesis of MPN is still poorly characterized. Akt is phosphorylated and activated via PI3K in response to signals originated by the erythropoietin (EPO) receptor; in particular, Akt is able to support erythroid differentiation in JAK2-deficient fetal liver progenitor cells through a mechanism downstream of EpoR [39] and at least in part related to GATA-1 phosphorylation [40]. Akt resulted strongly activated in erythroblasts from the bone marrow and the spleen of mice expressing a conditional *JAK2*V617F knock-in allele, especially in V617F homozygous animals [19]. Furthermore, phosphorylated STAT5 and Akt were found expressedat high levels in the bone marrow of MPN patients, particularly in megakaryocytes [41], consistent with the strong inhibition of human megakaryocyte progenitors by rapamycin [42]. Finally, inhibitors of the JAK/STAT and PI3K/Akt pathway caused comparable inhibition of EEC formation and EPO-induced erythroid differentiation in cultured progenitor cells of patients with PV [43]. All this evidence is in favor of abnormal Akt/mTOR signaling in MPN cells and constitute the basis for exploring the potential effectiveness of drugs targeting this pathway in MPN cells.

In this study we evaluated the effects of mTOR inhibitors, either as single drugs or in combination with JAK2 inhibitors, in different cellular models and primary cells from patients with MPN. We present evidences that drugs targeting mTOR signaling exert significant inhibition of MPN cells and their activity is synergistically enhanced by co-treatment with a JAK2 inhibitor. Therefore, these results reinforce the pathogenetic role of disregulated Akt/mTOR pathway in MPNs and open new avenues for the treatments of these disorders.

## Materials and Methods

### Reagents

RAD001 (a mTOR specific allosteric inhibitor with activity against TORC1) was provided by Novartis (Basel, CH); PP242 [36] (an ATP domain inhibitor of mTOR, with activity against TORC1 and TORC2) was obtained from Sigma-Aldrich (St. Louis, Germany). The JAK1/JAK2 kinase ATP-competitive inhibitors AZD1480 [44] and INC424 (ruxolitinib) were provided by D. Huszar (AstraZeneca, Waltham, MA, USA) and T. Radimeski (Novartis, Basel, CH), respectively. Antibodies against phospho(P)-STAT5 (Tyr694), STAT5, P-4E-BP1 (Thr70), 4E-BP1, P-JAK2 (Tyr1007/1008) and JAK2 were from Cell Signaling Technology (Danvers, MA, US). Anti-human tubulin antibody was from Santa Cruz Biotechnology (Santa Cruz, CA, US). Recombinant growth factors were purchased from Miltenyi Biotec (Gladbach, Germany).

### Cell Lines and Cell Culture

The *JAK2*V617F mutated HEL and SET2 human cell lines were purchased from the German Collection of Microorganisms and Cell Cultures (DSMZ, Braunschweig, Germany). Murine Ba/F3 and Ba/F3-EPOR cells expressing *JAK2* wild-type (wt) or *JAK2*V617F (VF) were donated by R. Skoda (University of Basel, Switzerland) [45]. Cell lines were cultured in RPMI 1640 supplemented with 10% fetal bovine serum (FBS; Lonza, Belgium) (20% for SET2 cells), antibiotics and L-glutamine. Recombinant human EPO (rhEPO; Sigma) was added to *JAK2* wt Ba/F3-EPOR cells, that require the cytokine for survival and proliferation, at final concentration of 1 U/mL. This concentration was chosen based on preliminary experiments showing that this amount of cytokine, in addition to support cell proliferation and survival (≥90% of cells were routinely viable in the cultures), promoted phosphorylation of STAT5 at such an extent that was very close to that measured in cultures of Ba/F3-EPOR VF cells maintained in a cytokine-free medium (**Figure S1**).

### Human Cells

Samples of peripheral blood or bone marrow were obtained from patients diagnosed with PV or PMF (2008 WHO criteria) [46] under a protocol approved by Institutional Review Board of Azienda Ospedaliera-Universitaria Careggi and after obtaining a written informed consent; CD34^+^ cells were immunomagnetically selected as described [47]. Control CD34^+^ cells were obtained from discarded cord blood units. Research was carried out according to the principles of Declaration of Helsinki.

### Inhibition of Proliferation Assay, Clonogenic Assay, and Apoptosis or Cell Cycle Analysis

Ba/F3-EPOR cells, both wt and VF, HEL and SET2 cells were plated at 2×10^4^ in 96-well culture tissue plates with increasing concentrations of the drug(s), in triplicate, and the amount of viable cells was assessed at 48 h using the WST-1 assay (Roche, USA) after normalization to wells containing an equivalent volume of vehicle (DMSO) only. For clonogenic assay, 5×10^3^ cells were plated in 0.5% agar in medium supplemented with 10% FBS (plus 1 U/mL EPO in case of Ba/F3-EPOR wt cells); variable amount of the drug(s) (or an equivalent volume of vehicle in control plates) was added once at the beginning of culture. Colonies were enumerated by inverted microscopy after 7 day incubation, in duplicate. Quantification of apoptotic cells was accomplished by flow cytometry using the Annexin-V-FLUOS Staining kit (Roche); at least 20,000 events were acquired. For cell cycle distribution analysis by flow cytometry, 1×10^6^ cells were treated with ethanol 95%, RNase 10 µg/mL and propidium iodide 50 mg/mL.

The concentration at which 50% inhibition (IC_50_) of cell proliferation or colony formation, promotion of apoptosis or change in distribution of the cells in cell cycle phase occurred was calculated using the Origin software (v7.5, OriginLab, Northampton, MA). In experiments where two drugs were concurrently administered, the combination index (CI), that is a measure of the interaction between two drugs, was calculated according to the median-effect principle of the Chou and Talalay method [48] using the CalcuSyn software (Biosoft Cambridge, UK). According to this formula, with CI<1 the interaction of two drugs is considered synergistic, when CI = 1 the interaction is additive, and when CI>1 the interaction is antagonistic [48].

### Colony Assay for Human Hematopoietic Progenitors and CD34^+^ Proliferation Assay

Bone marrow mononuclear cells from MPN patients or control subjects were plated at 1×10^5^/mL in methylcellulose (MethoCult; StemCell Technologies, Vancouver, Canada) supplemented with SCF 50 ng/mL, IL-3 10 ng/mL, IL-6 10 ng/mL, GM-CSF 10 ng/mL, G-CSF 10 ng/mL and EPO 1 U/mL for the growth of BFU-E and CFU-GM. For the growth of CFU-Mk, 5×10^4^/mL CD34^+^ cells were plated in a 24-well plate in Megacult Collagen and medium with lipids (StemCell Technol.) supplemented with Thrombopoietin 50 ng/mL, IL-3 10 ng/mL, IL-6 10 ng/mL. Colonies were enumerated on day 14 according to standard criteria. EEC assay was performed by plating 2.5×10^5^/mL peripheral blood mononuclear cells from PV patients in methylcellulose containing leukocyte-conditioned medium without EPO (StemCell Technol., cat. No.#04531); hemoglobinized colonies were scored at 10 days.

To measure the drug-induced inhibition of CD34^+^ cell growth, purified cells were plated at 3·10^4^ cells/well in IDMEM supplemented with cytokines and variable amounts of the drugs were added. Cell proliferation was evaluated using the WST-1 Assay (Roche, USA) after 48 h and results were normalized to wells containing vehicle only.

### SDS-PAGE Western Blotting

Cells were resuspended in RIPA lysis buffer (50 mM pH 7.4 Tris-HCl, 150 mM NaCl, 1% NP-40, 1 mMEDTA) containing a proteinase inhibitor cocktail (Halt Protease Inhibitor Cocktail Kit, PIERCE, Rockford, IL, US) and subjected to sodium dodecyl sulphate polyacrylamide gel electrophoresis separation and western blotting onto Immunoblot PVDF membrane (BioRad, Hercules, CA, US), according to standard protocols. Membranes were probed with primary antibodies followed by horseradish peroxidase-conjugated anti-Ig antibody produced in rabbits (Sigma-Aldrich); immunoreactive proteins were revealed with ECL using the Image Quant 350 apparatus (GE Healthcare, Little Chalfont, UK).

### RNA Isolation and Real-Time Quantitative PCR (RTQ-PCR)

Total RNA was purified using Trizol (Invitrogen-Life Technologies, Paisley, UK), and the RNA concentration and purity/integrity was determined with NanoDrop ND-1000 spectrophotometer (NanoDrop Techn., Wilmington, DE, USA). One microgram of RNA was reverse transcribed using High Capacity cDNA Archive Kit (Applied Biosystems, Foster City, CA). RT-QPCR reactions were performed with the TaqMan Universal PCR Master Mix using ABI PRISM 7300 HT and TaqMan® Gene Expression Assays (Applied Biosystems), in triplicate. Gene expression profiling was achieved using the Comparative cycle threshold (C_T_) method of relative quantitation using VIC-labeled *RNaseP* probe as the housekeeping gene (ΔC_T_).

### Statistical Methods

Comparison between groups was performed by the Mann-Whitney *U* or Fisher test as appropriate, using the SPSS (StatSoft, Inc., Tulsa, OK) or Origin software. The level of significance from two-sided tests was P<0.05.

## Results

### Inhibition of mTOR Signaling Results in Reduced Proliferation and Impaired Colony Formation of *JAK2*V617F Mutant Cell Lines

**Figure 1:**
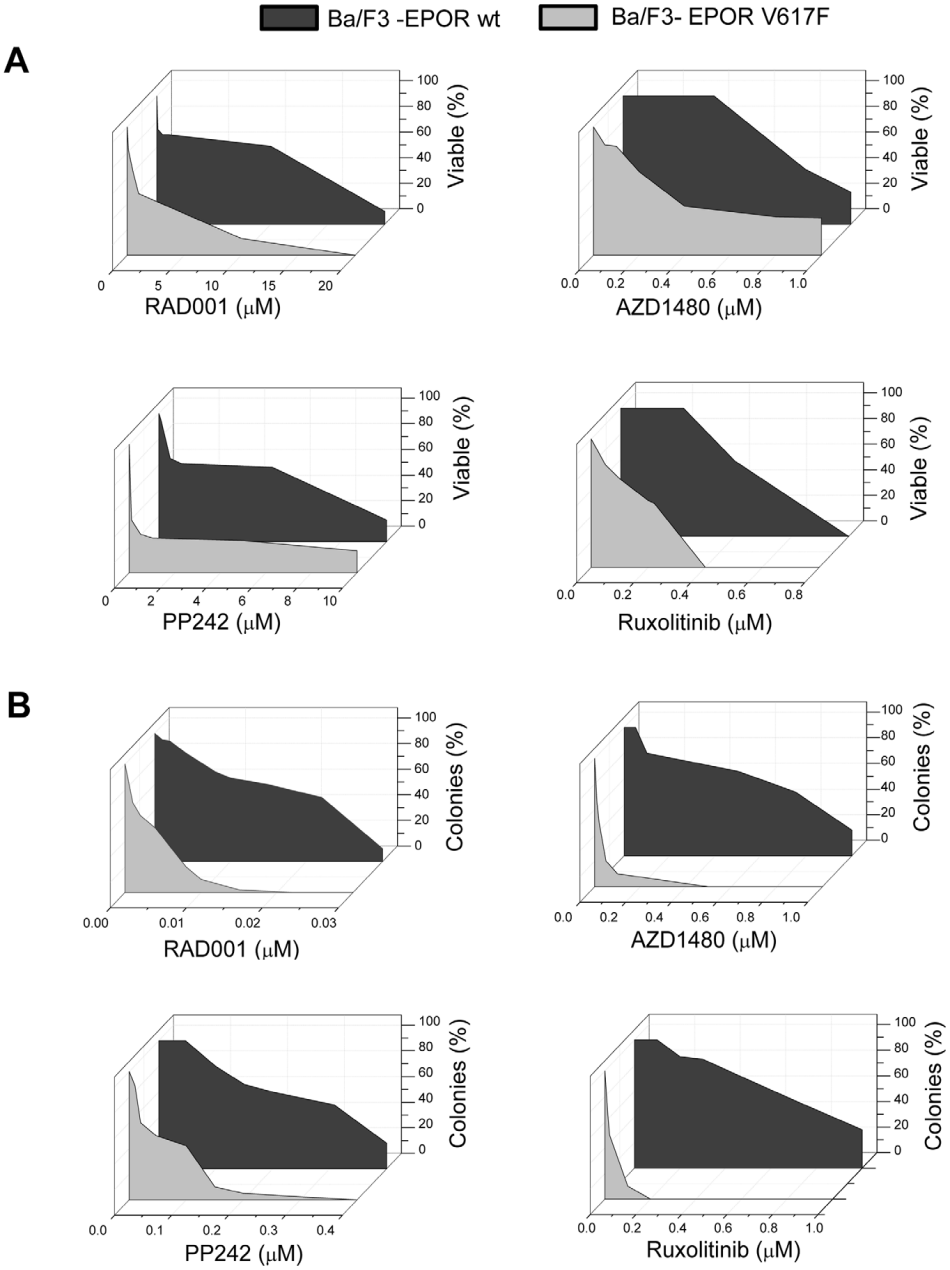
Inhibition of cell proliferation and colony formation of wild-type (wt) and JAK2V617F (VF)-expressing Ba/F3-EPOR cells by mTOR and JAK2 inhibitors. Results of short-term proliferation assay (panel A) and clonogenic assay (panel B) are shown. Values represent the percent inhibition compared to control wells containing vehicle only. Data shown are the mean of at least five (panel A) and three (panel B) independent experiments. Individual IC_50_ values are reported in the Results section.

To ascertain the effects of mTOR inhibition on the growth of cell lines harboring the *JAK2*V617F mutation we employed the selective allosteric mTOR inhibitor RAD001 and the ATP competitive inhibitor of the active site of mTOR, PP242. Concurrently, we used the two ATP-competitive JAK2 inhibitors ruxolitinib and AZD1480. *JAK2* wt Ba/F3 cells engineered to express the erythropoietin receptor (Ba/F3-EPOR wt) and the cytokine-independent counterpart with ectopic expression of *JAK2*V617F (Ba/F3-EPOR VF) were exposed to increasing drug concentrations in proliferation (48 h; Figure 1A) and clonogenic (7 days; Figure 1B) assay. We found that Ba/F3-EPOR VF cells were definitely more sensitive to inhibition of the mTOR pathway than the wt counterpart: the IC_50_ (mean±SD) was 651±50 nM versus 11,000±1,000 nM and 500±100 nM versus 5,931±1,000 nM, respectively, in case of RAD001 and PP242 (P<0.001 for both) (Figure 1A). Similarly, JAK2 inhibitors effectively prevented the growth of Ba/F3 VF cells with greater sensitivity compared to wt cells; the IC_50_ was 313±23 nM versus 752±30 nM and 220±20 nM versus 457±15 nM in case of AZD1480 and Ruxolitinib, respectively (P<0.001 for both) (Figure 1A). Also the clonogenic potential of Ba/F3-EPOR VF cells, evaluated in semisolid cultures, was potently inhibited by mTOR and JAK2 inhibitors at very low nanomolar concentrations, as shown in Figure 1B. The IC_50_ for VF and wt Ba/F3-EPOR cells was 4±2 nM versus 22±10 nM and 47±12 nM versus 308±100 nM for RAD001 and PP242, respectively, and 19±11 nM versus 750±100 nM and 20±15 nM versus 740±100 nM for AZD480 and ruxolitinib, respectively (P<0.01 for all combinations). Similar results were obtained using the parental IL-3- dependent Ba/F3 JAK2 wt and VF cell lines (not shown). Finally, we tested the effect of different inhibitors also in liquid cultures of cytokine-independent Ba/F3 VF cells maintained in the presence of Epo (1 U/mL). We found that the IC_50_ remained significantly lower (1,231±100 nM for RAD001 and 750±100 nM for PP242) than wt cells (11,000±1,000 nM and 5,931±1,000 nM, respectively; P<0.01), reinforcing the greater sensitivity of VF cells to mTOR inhibitors. On the other hand, the addition of Epo largely prevented the inhibitory effects of JAK2 inhibitors: the resulting IC_50_ was 707±11 nM for AZD1480 and 521±45 nM for ruxolitinib compared with 752±30 nM and 457±15 nM, respectively, in wt cells.

We then evaluated the effect of increasing drug amounts on the rate of cell division through the analysis of changes in cell cycle of Ba/F3-EPOR VF cells that could contribute to slowed cell division observed in previous experiments (Figure S2A). After 24 hours, mTOR and JAK2 inhibitors induced a block in the G0/G1 phase with subsequent decrease in the S-phase: a 50% reduction of the cells in the S-phase compared to untreated cells was reached at a median value of 6.5 µM for RAD001, 1.87 µM for PP242, 1.2 µM for AZD1480 and 1.87 µM for Ruxolitinib. On the other hand, the proportion of Annexin V positive apoptotic cells increased dose-dependently with PP242 and both the JAK2 inhibitors: the median IC_50_ values for apoptosis were 4.0 µM for PP242, 0.8 µM for AZD1480 and 1.3 µM for Ruxolitinib (Figure S2B). In cells exposed to RAD001 an increase of apoptotic cells became appreciable only at drug concentrations >3.3 µM followed by a plateau, resulting in an IC_50_ value of 5.0 µM, indicating modest efficacy of RAD001 as inducer of cell apoptosis.

Efficacy of the drugs was also assessed in the human *JAK2*V617F mutated HEL and SET2 cell lines. We found that both cells lines were relatively insensitive to RAD001 showing an IC_50_ in the micromolar range (mean value, 14.0 µM and 17.0 µM, respectively for HEL and SET2 cells), unlike the case of PP242 and JAK2 inhibitors that efficaciously prevented cell proliferation at nanomolar concentration (see Table 1). On the other hand, counterintuitively, colony formation by HEL and SET2 cells was efficaciously prevented at nanomolar concentrations of RAD001 (mean value, 0.93 nM and 0.044 nM for HEL and SET2 cells, respectively) and PP242 (0.172 nM and 0.062 nM), and similar for JAK2 inhibitor AZD1480 (0.46 nM and 0.035 nM) and Ruxolitinib (0.37 nM and 0.027 nM) (Table 1). We are not able to provide obvious explanations for such contrasting findings concerning the efficacy of RAD001 in clonogenic versus proliferation assay, that could not be actually ascribed to different culture conditions, variable times of drug exposure, types of plastics used as well as a number of other variables we deliberately introduced in the system. One possibility might be that optimal inhibition of HEL and SET2 cell proliferation by RAD001 requires some kind of cell-to-cell interactions that are favoured in the semisolid media as compared with liquid cultures. These experiments also indicated that HEL cells, that display multiple copies of *JAK2*V617F, resulted significantly less sensitive than the *JAK2*V617F heterozygous SET2 cells.

**Table 1:** Effects of mTOR (RAD001 and PP242) and JAK2 (Ruxolitinib and AZD1480) inhibitors on the proliferation rate and clonogenic growth of human *JAK2*V617F mutated cell lines.

	IC50 (µM)			
	RAD001	PP242	AZD1480	Ruxolitinib
Proliferation assay				
HEL	14±2.8	1.5±0.1**	0.86±0.02**	0.79±0.15**
SET2	17.0±0.3	0.28±0.01	0.09±0.005**	0.16±0.02**
Clonogenic assay				
HEL	0.93±0.01**	0.172±0.059**	0.46±0.03**	0.37±0.177**
SET2	0.044±0.015**	0.062±0.019**	0.035±0.01**	0.027±0.009**

In further experiments aimed at evaluating the effects of treatment on cell cycle and apoptosis we used SET2 cells as a model. Mirroring the results obtained in Ba/F-EPOR VF cells, we observed a dose-dependent decrease of SET2 cells in the S-phase due to a block in G0/G1: a 50% reduction of SET2 cells in the S-phase compared to controls was obtained with a median concentration of 33 µM for RAD001, 2.7 µM for PP242, 1.5 µM for AZD1480 and 10 µM for Ruxolitinib. Apoptosis was promoted more efficaciously by JAK2 inhibitors (median IC_50_ values for apoptosis were 0.4 µM for AZD1480 and 0.5 µM for Ruxolitinib) than RAD001 (38 µM) and PP242 (5.2 µM) (data not shown in detail).

Inhibition of the mTOR- and JAK2-dependent signalling pathways in Ba/F3-EPOR and SET2 cells exposed to the relevant drugs was supported by results of western blot experiments showing reduced levels of phosphorylated 4eBP1, in case of mTOR inhibitors, and phosphorylated JAK2 and STAT5 in case of JAK2 inhibitors (Figure S3A, B). We also assayed the mRNA level of selected downstream targets in SET2 cells We found that mTOR and JAK2 inhibitors similarly induced statistically significant early downregulation of *CCND1*, that however resulted more durable (at 24 h) with JAK2 inhibitors. On the other hand, JAK2 inhibitors only caused a statistically significant downregulation of antiapoptotic genes such as *PIM1* and *BCLX_L_* (Figure S4).

As a whole, these data indicate that blocking mTOR signalling pathway is effective in reducing proliferation and clonogenic potential of cells expressing the *JAK2*V617F mutation; however, mTOR inhibitors appeared to exert mainly a cytostatic rather than an apoptotic effect when compared with JAK2 inhibitors.

### mTOR Inhibitors Impair the Proliferation and Clonogenic Potential of CD34^+^ Cells from MPN Patients

**Figure 2:**
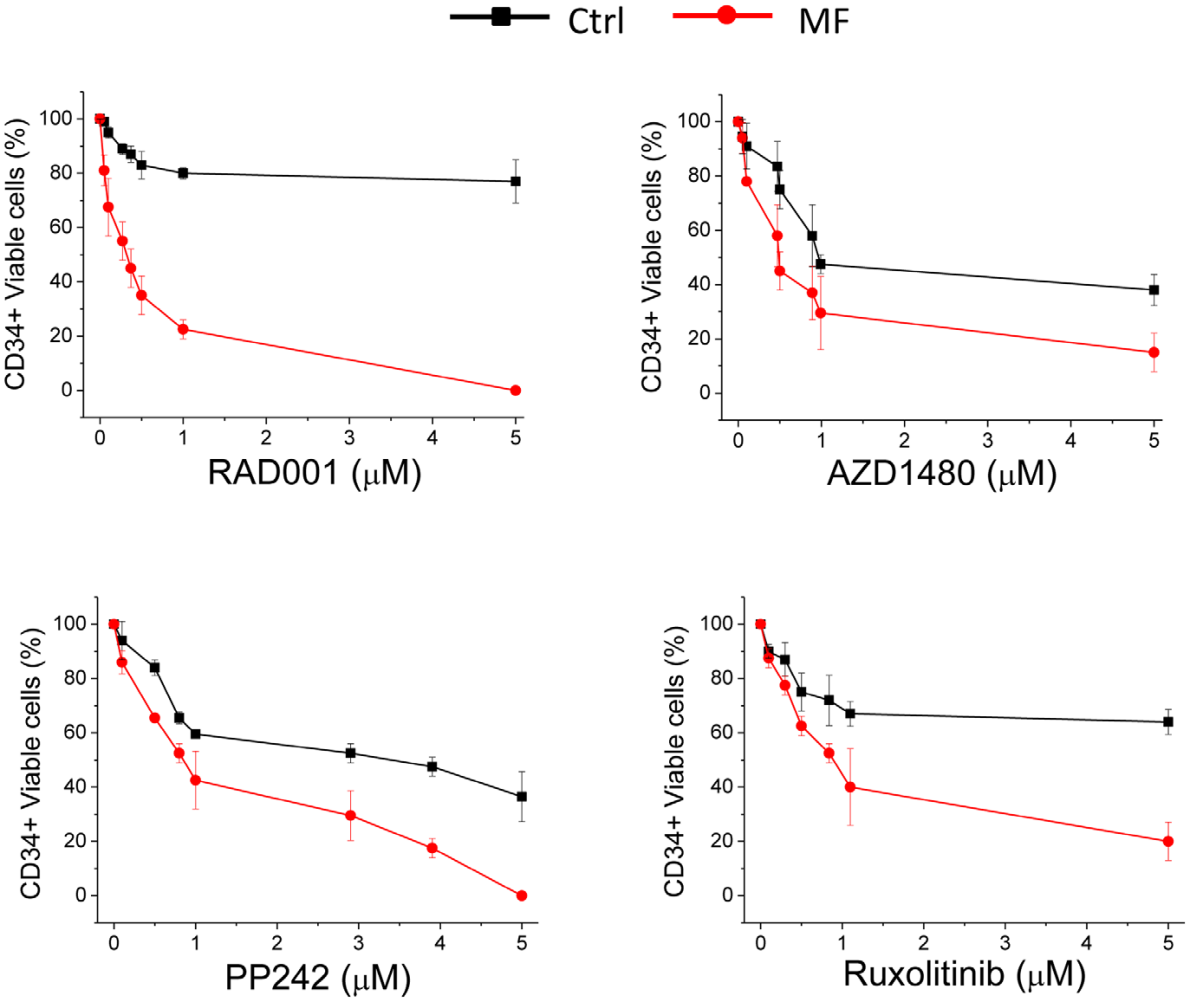
Inhibition of proliferation of CD34^+^ cells from PMF patients by mTOR and JAK2 inhibitors. CD34^+^ cells from patients with PMF (n = 6) or control subjects (Ctr; n = 5) were plated at 2×10^5^/mL in 96-well culture tissue plates with increasing concentrations of the drugs, in triplicate; viable cells were assessed after 48 hrs using the WST-1 assay and normalized to wells containing an equivalent volume of vehicle (DMSO) only. Values shown are the mean±SD The concentration at which 50% inhibition of proliferation occurred (IC_50_) was calculated (see Results section for details).

To exploit whether the proliferation of primary MPN cells could be similarly affected by mTOR inhibitors, we incubated CD34^+^ cells from PMF patients and healthy controls (n = 6 and n = 5, respectively) with increasing concentration of RAD001 and PP242, and measured the proportion of viable cells relative to control wells containing vehicle only; an optimized cytokine cocktail was added to both patients’ and controls’ wells to allow cell survival. As shown in Figure 2, the proliferation of PMF CD34^+^ cells was inhibited at significantly lower concentration of RAD001 (IC_50_ = 271±100 nM) and PP242 (IC_50_ 800±200 nM) compared to healthy subjects (IC_50_ = >5,000 nM and 2,900±1,000 nM, respectively (P<0.01 for both). Similarly AZD1480 and ruxolitinib produced more effective inhibition of the proliferation rate of CD34^+^ cells from PMF patients (IC_50_ = 474±30 nM and 840±300 nM, respectively) compared to control cells (IC_50_ = 891±100 nM and >5,000 nM; P<0.05 and 0.001, respectively).

**Figure 3:**
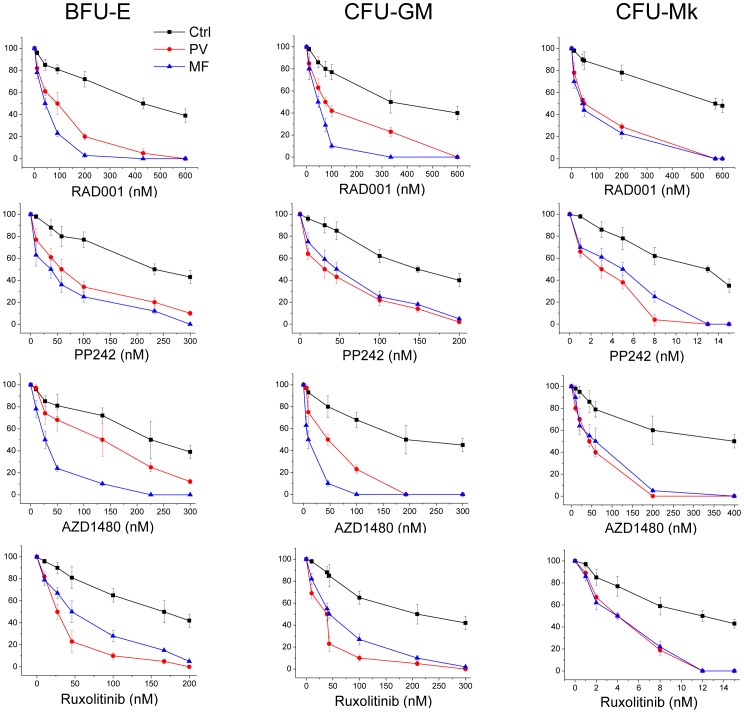
mTOR and JAK2 inhibitors preferentially suppress colony formation from hematopoietic progenitors of MPN patients as compared to healthy controls. Mononuclear cells from patients with PV or PMF (n = 10 each) or control subjects (n = 10) were plated at 10^5^/mL in cytokine-supplemented methylcellulose medium for the growth of erythroid burst-forming unit (BFU-E) and granulocyte-macrophage colony-forming unit (CFU-GM), and CD34^+^ cells (5×10^4^/mL) were plated in collagen-based medium for megakaryocyte colony-forming unit (CFU-Mk). Increasing amounts of mTOR inhibitors (RAD001 and PP242) or JAK2 inhibitors (AZD1480 and Ruxolitinib) were added at the beginning of culture. Colonies were enumerated on day 14 and expressed as a percentage of the colonies grown in control plates containing vehicle only.

Then, to evaluate the effects of mTOR inhibitors on the colony-forming activity of hematopoietic progenitors from MPN patients, bone marrow mononuclear cells or CD34^+^ cells from PMF and PV patients and healthy controls were cultured in the presence of cytokines optimally supporting the growth of BFU-E, CFU-G/GM or CFU-Mk. A separate set of experiments was also devised to evaluate drugs’ effect on the formation of erythropoietin endogenous erythroid colonies (EEC) in patients with PV. Drugs were added at increasing concentrations once at the beginning of culture and the IC_50_ was established by comparison to control dishes containing vehicle only. As shown in Figure 3, colony formation from MPN patients was dose-dependently reduced by mTOR inhibitors; the data reported in Table 2 indicate that MPN progenitor growth could be inhibited with IC_50_ drug concentration significantly lower than control cells (in case of RAD001, from 4.7 to 13-fold lower and in case of PP242 from 2.6 to 6.1-fold lower, depending on colony type), pointing to a substantial degree of drug selectivity against MPN cells. Progenitors forming megakaryocytic colonies were the most sensitive. Results obtained with JAK2 inhibitors were similar, with IC_50_ values lower than control of 1.6–21.4-fold in case of AZD1480 and 3–6.2-fold in case of Ruxolitinib. By keeping separate *JAK2*V617F positive and negative PMF subjects (n = 5 each) we did not observe appreciable difference in the inhibitory effect of either drugs depending on the *JAK2* mutation status of the patients (not shown in detail). Finally, we determined that the growth of EEC from circulating progenitors in PV patients (n = 5) could be inhibited at very low nanomolar concentrations of RAD001 (IC_50_ = 15.0±10.0 nM), PP242 (IC_50_ = 1.0±0.7 nM), AZD1480 (IC_50_ = 19.0±2.0 nM) and Ruxolitinib (IC_50_ = 1.8±1.0 nM), indicating a high sensitivity of these progenitors, that are considered as being mostly *JAK2*V617 mutated [10], [49], to both mTOR and JAK2 inhibitors.

**Table 2:** Activity of mTOR (RAD001 and PP242) and JAK2 (Ruxolitinib and AZD1480) inhibitors on the clonogenic potential of hematopoietic progenitors from patients with PV and PMF (n = 10 each) and healthy controls (n = 10).

IC_50_ (nM)				
	RAD001	PP242	AZD1480	Ruxolitinib
**BFU-E**				
Ctrl	432±22	233±50	226±17	167±70
PV	91±15**	58±18*	135±19**	27±7*
MF	42±10**	38±8*	27±8**	46±10*
**CFU-GM**				
Ctrl	335±16	148±40	193±13	209±40
PV	75±4 **	31±15*	46±5**	39±6*
MF	46±13**	46±20*	9±3**	43±4*
**CFU-Mk**				
Ctrl	572±13	13±2	400±12	12±5
PV	50±5**	3±1**	44±2**	4±2*
MF	44±5**	25±2 **	59±3**	4±3*

The IC50 was calculated using data derived from the sensitivity curves to drugs reported in Figure 3. *, P<0.05, **, P<0.01.

In aggregate, these data indicate that colony growth from hematopoietic progenitors of MPN patients can be efficiently and selectively inhibited by mTOR inhibitors compared to controls.

### Combination of mTOR and JAK2 Inhibitor Results in Improved Efficacy Against MPN Cells

Next, we evaluated the effects of concurrent inhibition of mTOR and JAK2 signaling in Ba/F3-EPOR VF and SET2 cells. Cells were put in culture using progressively increasing concentrations of the drugs and the proportion of viable cells at 48 h, or the number of colony formed at 10d, was measured; the combination index (CI), as a measure of the interaction between two drugs, was calculated as detailed in the Methods section (examples are shown in Figure S5 and 6). Results presented in Table 3 indicate that combination of a mTOR and JAK2 inhibitor resulted in markedly lower concentrations of the individual drugs necessary to produce a 50% inhibition in the assay. For example, the proliferation of SET2 cells could be efficaciously inhibited with RAD001 amounts of 9.4 to 10.6-fold lower, 2.3 to 7.9-fold in case of PP242, 3.1 to 9.4-fold in case of AZD1480 and 3.0 to 5.1-fold in case of Ruxolitinib, depending on the drug combinations. The calculated CI values was always lower than 1, ranging from 0.33 to 0.98, to indicate synergism of the mTOR and JAK2 inhibitors in the two cell lines and experimental settings.

**Table 3:** Combining mTOR and JAK2 inhibitor resulted in synergistic inhibition of the proliferation rate (A) and clonogenic potential (B) of Ba/F3-EPOR VF and SET2 cells.

Single drug					Drug combination				
IC_50_ (nM)					IC_50_ (nM)			CI index	
**Ba/F3-EPOR VF**									
**RAD001**	**AZD1480**				**RAD001**	**AZD1480**			
651±50	313±23				121±39	58±19			**0.5±0.1**
**RAD001**	**Ruxolitinib**				**RAD001**	**Ruxolitinib**			
651±50	220±20				304±83	72±24			**0.3±0.1**
**PP242**	**AZD1480**				**PP242**	**AZD1480**			
500±100	313±23				212±18	164±32			**0.5±0.2**
**PP242**	**Ruxolitinib**				**PP242**	**Ruxolitinib**			
500±100	220±20				265±190	94±38			**0.79±0.3**
**SET2**									
**RAD001**		**AZD1480**	**RAD001**				**AZD1480**		
17,000±3,000		90±5	1,798±87				9.5±0.4		**0.48±0.05**
**RAD001**		**Ruxolitinib**	**RAD001**				**Ruxolitinib**		
17,000±3,000		160±24	1,597±1,000				53±35		**0.66±0.05**
**PP242**		**AZD1480**	**PP242**				**AZD1480**		
285±11		90±5	122±22				29±26		**0.56±0.1**
**PP242**		**Ruxolitinib**	**PP242**				**Ruxolitinib**		
285±11		160±24	36±32				31±21		**0.27±0.2**
**Ba/F3-EPOR VF**									
**RAD001**	**AZD1480**				**RAD001**	**AZD1480**			
4±2	19±11				0.9±0.4	4.3±2.2			**0.4±0.3**
**RAD001**	**Ruxolitinib**				**RAD001**	**Ruxolitinib**			
4±2	20±15				1.1±0.2	5.9±1.9			**0.6±0.3**
**PP242**	**AZD1480**				**PP242**	**AZD1480**			
500±100	19±11				16±4	6.5±1.8			**0.8±0.2**
**PP242**	**Ruxolitinib**				**PP242**	**Ruxolitinib**			
500±100	20±15				6.2±5	2.7±2			**0.24±0.2**
**SET2**									
**RAD001**		**AZD1480**		**RAD001**			**AZD1480**		
44±15		35±10		5.2±4			4.1±3		**0.23±0.1**
**RAD001**		**Ruxolitinib**		**RAD001**			**Ruxolitinib**		
44±15		27±9		6.2±1			3.7±0.4		**0.32±0.04**
**PP242**		**AZD1480**		**PP242**			**AZD1480**		
62±19		35±10		16±4			1±0.5		**0.4±0.05**
**PP242**		**Ruxolitinib**		**PP242**			**Ruxolitinib**		
62±19		27±9		29±16			12.8±7		**0.9±0.13**

Data shown (mean ± SD) are from at least two experiments. The Combination Index (CI) was calculated as described in Materials and Methods. A CI<1 indicates that the interaction of the two drugs is synergistic. The first two columns report, for convenience, the IC_50_ value of the individual drugs in these experimental settings (see Results section for details).

We finally assessed the efficacy of combining mTOR and JAK2 inhibitor in preventing the generation of EEC from peripheral blood mononuclear cells of PV patients. As reported in Table 4, the concentration of mTOR and JAK2 inhibitors required to produce a 50% inhibition of EEC were reduced, as compared to each drug alone, of 5.2 to 7.9-fold in case of RAD001, 2.5 to 6.2-fold in case of PP242, 7.6 to 27-fold in case of AZD1480 and 6 to 9-fold in case of Ruxolitinib. The calculated CI ranged from 0.13 to 0.63.

**Table 4:** Combination of mTOR and JAK2 inhibitor resulted synergistic in reducing erythropoietin-independent colony formation in primary cells from PV patients.

		Drug combination		
IC_50_ (nM)		IC_50_ (nM)		CI index
**RAD001**	**AZD1480**	**RAD001**	**AZD1480**	
15±10	19±2	2.9±2	2.5±1	**0.61±0.2**
**RAD001**	**Ruxolitinib**	**RAD001**	**Ruxolitinib**	
15±10	1.8±1	1.9	0.2	**0.26**
**PP242**	**AZD1480**	**PP242**	**AZD1480**	
1±0.7	19±2	0.04	0.7	**0.13**
**PP242**	**Ruxolitinib**	**PP242**	**Ruxolitinib**	
1±0.7	1.8±1	0.16±0.1	0.3±0.2	**0.5±0.3**

Data shown (mean ± SD) are derived from the analysis of two patients, in duplicate. The Combination Index (CI) was calculated as described in Materials and Methods. A CI<1 indicates that the interaction of the two drugs is synergistic. The first two columns (in gray) report, for convenience, the IC_50_ value of the individual drugs in this experimental setting (see Results section for details).

Therefore, these results strongly support synergism of mTOR and JAK2 inhibitors in inhibiting the growth of MPN cells.

## Discussion

Dysregulation of the JAK2/STAT pathway represents a central mechanism in the pathogenesis of MPNs: in fact, (i) the *JAK2*V617F gain-of-function mutation occurs in the majority of patients with PV and 60% of PMF and essential thrombocythemia [2], [3], (ii) other mutations (*MPL, LNK, CBL*) found in 5–10% can similarly activate the JAK/STAT pathway [5]–[8], [50], (iii) mouse models indicate that those mutations are able to induce a myeloproliferative disorder [11]–[19], (iv) the JAK/STAT pathway is involved in the dysregulated cytokine expression that accompanies MPNs and underlies some tract of the clinical phenotype [51], and finally (v) targeting activated JAK2 with ATP-competitive JAK2 inhibitors resulted in measurable clinical improvements in patients with myelofibrosis [25], [26]. The clinical efficacy of JAK2 inhibitors has been ascribed to a variable degree of myelosuppression and a general down-regulation of inflammatory cytokine signalling (at least in part mediated by the concomitant anti-JAK1 properties of some JAK2 inhibitors such as ruxolitinib) [52], well keeping in mind that none of the available molecules is specific to mutant as opposed to wild-type JAK2. Conceivably, in contrast to the capacity of JAK2 inhibitors to reduce the enlarged spleen and improve disease symptomatic manifestations, changes in the burden of mutated cells have been variable (with SAR302503 being reported as the most effective until now [53]) but usually modest, at least in the short-term follow-up. Also, while generally well tolerated, JAK2 inhibitors caused some degree of unwanted myelosuppression, particularly anemia and thrombocytopenia. Therefore, current efforts are directed towards additional targets involved in the dysregulated proliferation of MPN cells and/or novel therapeutic options with the hope to maximize anticancer efficacy and/or improve the tolerability profile of available JAK2 inhibitors.

In this study we have focused on the mammalian target of rapamycin (mTOR), a key downstream target of the PI3K/Akt pathway, with the objective to characterize the efficacy of mTOR inhibitors in different cellular models of MPNs, including primary cells. We found that *JAK2*V617F mutated human and murine leukemia cell lines are sensitive to mTOR inhibitors showing a dose-dependent inhibition of cell proliferation and clonogenic potential that mainly reflected a cytostatic rather than an apoptotic effect. However, the ATP mimetic inhibitor PP242 resulted a more potent and dose-dependent inducer of apoptosis than the allosteric inhibitor RAD001, leading to speculate that such a difference may be attributable to the activity of PP242 against both mTORC complexes as opposed to the inhibition of mTORC1 only exerted by RAD001 [31]; further experiments are in progress at this regard. We also showed that mTOR inhibitors inhibited the proliferation of CD34^+^ cells and hematopoietic colony formation from MPN patients at doses significantly lower than healthy subjects and potently reduced the generation of erythroid-independent colonies (EEC) that are considered to closely represent the MPN clone since they are mostly *JAK2*V617F mutated [10], [49]. As a whole, these results, indicating sensitivity of MPN cells to mTOR inhibitors, provided mechanistic explanation for the findings of a phase I/II trial that showed efficacy of RAD001 (Everolimus) against splenomegaly and symptomatic burden in patients with myelofibrosis [54] and reinforce the rationale for designing clinical trials with novel, and possibly more effective, drugs targeting the activated PI3K/Akt/mTOR pathway.

In fact, the abnormal activation of mTOR in cancer cells and its role in many critical cellular processes, together with the availability of a growing number of molecules entering the clinical scenario, makes it an attractive target for therapy in neoplasia where involvement of the mTOR pathway contributes to disease pathogenesis; activation of this pathway has been demonstrated in MPN cells [29], [30], *JAK2*V617F-expressing mice [19] [38] and primary samples [41], [43]. However, in most trials performed in different clinical settings [55] RAD001 and other rapalogs resulted in disease stabilization rather than tumor regression, an effect largely attributable to the predominantly cytostatic effect of these agents. Indeed, in a mouse myeloproliferative disease model characterized by constitutively active STAT5, treatment with rapamycin effectively reduced myeloid cell proliferation in transplanted mice and significantly prolonged survival; however, the myeloproliferative disease recurred once the treatment was stopped [56]. Such prevalent antiproliferative rather than pro-apoptotic effect of rapamycin derivatives including RAD001 has prompted on one side studies of combination with agents that preferentially induce apoptosis or are directed against other disease-associated targets [55] on the other the development of novel ATP-competitive mTOR inhibitors or dual PI3K/mTOR inhibitors. Bearing this in mind, we explored the effects of combining mTOR and JAK1/JAK2 inhibitors *in vitro*. We first determined that, in the same experimental settings, JAK2 inhibitors (AZD1480 [44] and Ruxolitinib [21]) efficaciously impaired the growth of *JAK2*V617F mutated mouse and human cell lines and primary cells by slowing the progression to S-phase of the cell cycle and exerting a more definite apoptotic effect, at least in part mediated by downregulation of BcLxL and PIM. Accordingly, we determined that combination of mTOR and JAK2 inhibitor resulted in significant synergism concerning the inhibition of proliferation and colony formation of mouse and human *JAK2*V617 mutated leukemia cell lines and prevented at very low nanomolar concentration the formation of EPO-independent erythroid colonies.

Combinations of mTOR inhibitor with chemotherapeutics, targeted drugs or antibodies are being explored in preclinical models and/or have been preliminary reported in clinical trials. At this regard, combination of rapamycin with ABT-737, an inhibitor of Bcl-X_L_, has recently been shown to provoke synergistic effects in mice with a myeloproliferative disorder due to constitutively active STAT5 and persistent activation of Akt/mTOR signalling [57]. Present data describing synergistic activity of mTOR- and JAK/STAT-directed therapies in cellular models of MPN foresee the opportunity of testing this association in clinical trials, with the expectation that such combination might results in a better therapeutic index producing more effective inhibition of clonal cells and also, due to the modest toxicity of mTOR inhibitors against normal cells, by reducing unwanted side effects of JAK2 inhibitors administered at lower, yet effective, dose.

## Supporting Information

Figure S1 **Effect of different amount of EPO added to the culture medium of Ba/F3-EPOR wild-type cells on the level of phosphorylated STAT5.** In order to determine the optimal amount of EPO that induced phosphorylation of STAT5 in Ba/F3-EPOR wild-type (wt) cells at level comparable with *JAK2*V617F (VF) mutated cells, we added different amounts of EPO and then processed the cells for western blot analysis after 6 h. Each phosphoSTAT5 band was first normalized by densitometric analysis using the ImageQuant 350 apparatus (GE Healthcare, Little Chalfont, UK) and the ImageQuant 5.2 software against corresponding total STAT5, then the values measured in wt cells were normalized against the level measured in VF cells considered as = 1. The amount of phosphoSTAT5 in the presence of 1 U/mL Epo was 1.2, close to VF cells.
(TIF)

Figure S2 **Effect of mTOR (RAD001 and PP242) and JAK2 (AZD1480 and Ruxolitinib) inhibitors on apoptosis and cell cycle in Ba/F3-EPOR VF cells.** In panel **A**, the frequency of cells in the G0/G1, S and M phase of the cell cycle was measured by flow cytometry after propidium iodide staining of Ba/F3-EPOR VF cells that had been exposed to the drugs for 18 h, and expressed as percent change compared to control cells (Ctrl) with vehicle. Results shown are the Mean±SD of at least three experiments. In panel **B**, the percentage of Annexin V-positive cells was measured in cultures of Ba/F3-EPOR VF cells exposed to varying amount of the drugs for 48 h; percentage was calculated after subtraction of background apoptosis in cells incubated without drug. Results shown are the Mean±SD of at least three experiments.
(TIF)

Figure S3 **Effect of mTOR (RAD001 and PP242) and JAK2 (AZD1480 and Ruxolitinib) inhibitors on key targets in Ba/F3-EPOR VF and SET-2 cells. A.** JAK2 wt and VF Ba/F3-EPOR cells were incubated for 6 h with final concentrations of the drugs corresponding to the IC_50_ values measured in proliferation assay (see Results for details). **B.** SET-2 cells were incubated for 24 h with increasing concentrations of the drugs, as indicated. The level of total and phosphorylated JAK2, STAT5, and 4EBP1 was analyzed by western blot. Tubulin was used for loading normalization. One representative of two to four similar experiments.
(TIF)

Figure S4 **Effect of mTOR (RAD001 and PP242) and JAK2 (AZD1480 and Ruxolitinib) inhibitors on the expression of selected downstream molecules in SET-2 cells.** The expression level of mRNA for PIM1, BCLxL and CCND1 was measured by RTQ-PCR in SET-2 cells at different times of incubation with the IC_50_ concentration of each drug, in triplicate. Gene expression profiling was achieved using the Comparative cycle threshold (CT) method of relative quantitation using VIC-labeled RNaseP probe as PIM1the housekeeping gene (ΔCT). Where not indicated, SD was <10% for each experimental point and was not depicted. This experiment has been repeated twice with similar results. *, P<0.05; **, P<0.01.
(TIF)

Figure S5 **Analysis of the combinatory effects of mTOR (RAD001 and PP242) and JAK2 (AZD1480 and Ruxolitinib) inhibitors in Ba/F3-EPOR VF cells.** Cells were evaluated in proliferation assay for 48 h using WST-1 assay to measure the proportion of cells alive, after subtraction of the value measured in control wells with vehicle only. “Fa” is the cell fraction affected by the dose of the drug; the combination Index (**CI**) was calculated as described in Materials and Methods using Calcusyn Software. A CI<1 indicates that the interaction of the two drugs is synergistic.
(TIF)

Figure S6 **Analysis of the combinatory effects of the mTOR (RAD001 and PP242) and JAK2 (AZD1480 and Ruxolitinib) inhibitors in SET-2 cells.** Cells were evaluated in proliferation assay for 48 h using WST-1 assay to measure the proportion of cells alive, after subtraction of the value measured in control wells with vehicle only. “Fa” is the cell fraction affected by the dose of the drug; the combination Index (**CI**) was calculated as described in Materials and Methods using Calcusyn Software. A CI<1 indicates that the interaction of the two drugs is synergistic.
(TIF)
