# Supplementary figures and images for: mTOR Inhibitors Alone and in Combination with JAK2 Inhibitors Effectively Inhibit Cells of Myeloproliferative Neoplasms
Source: PLoS One. 2013 Jan 31;8(1):e54826. doi: 10.1371/journal.pone.0054826 (PMC3561413; doi:10.1371/journal.pone.0054826)

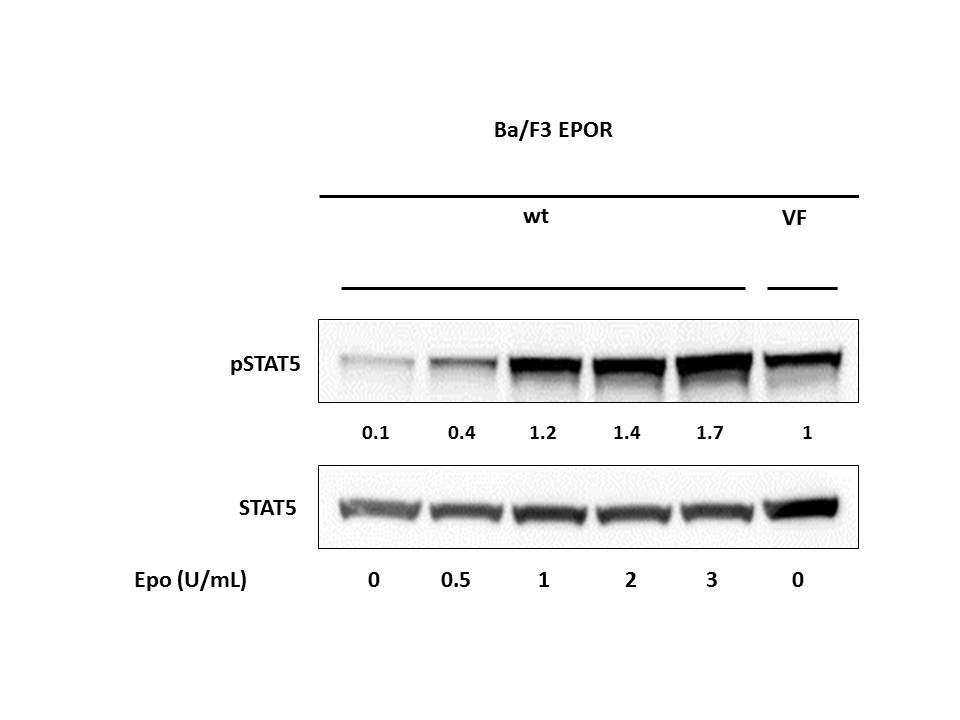

Supplement: Figure S1 — Effect of different amount of EPO added to the culture medium of Ba/F3-EPOR wild-type cells on the level of phosphorylated STAT5. In order to determine the optimal amount of EPO that induced phosphorylation of STAT5 in Ba/F3-EPOR wild-type (wt) cells at level comparable with JAK2V617F (VF) mutated cells, we added different amounts of EPO and then processed the cells for western blot analysis after 6 h. Each phosphoSTAT5 band was first normalized by densitometric analysis using the ImageQuant 350 apparatus (GE Healthcare, Little Chalfont, UK) and the ImageQuant 5.2 software against corresponding total STAT5, then the values measured in wt cells were normalized against the level measured in VF cells considered as = 1. The amount of phosphoSTAT5 in the presence of 1 U/mL Epo was 1.2, close to VF cells. (TIF) [file pone.0054826.s001.tif]

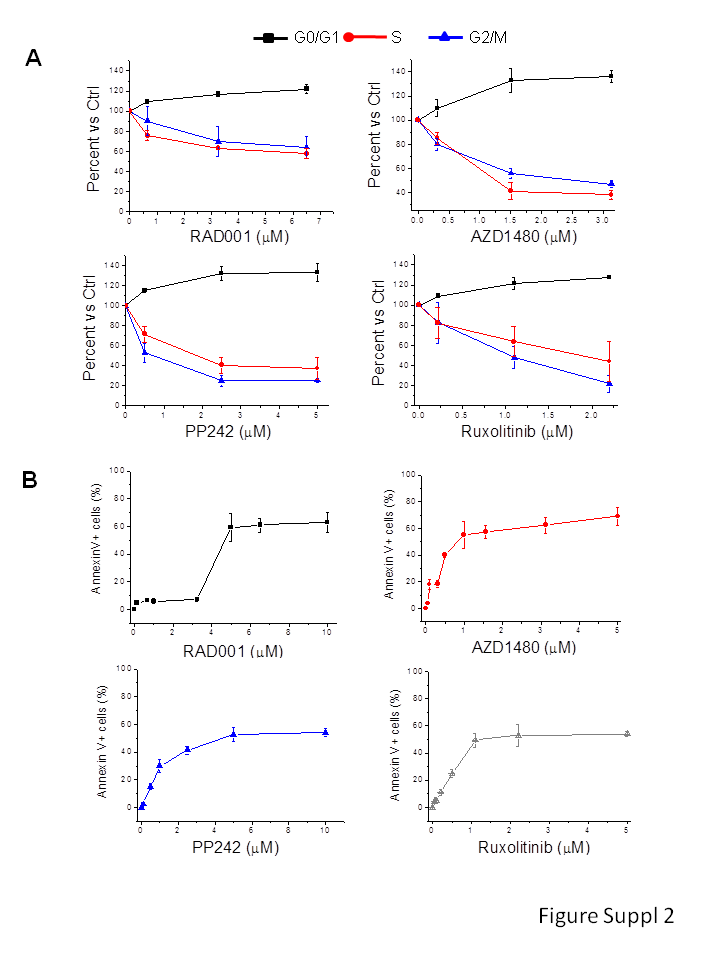

Supplement: Figure S2 — Effect of mTOR (RAD001 and PP242) and JAK2 (AZD1480 and Ruxolitinib) inhibitors on apoptosis and cell cycle in Ba/F3-EPOR VF cells. In panel A, the frequency of cells in the G0/G1, S and M phase of the cell cycle was measured by flow cytometry after propidium iodide staining of Ba/F3-EPOR VF cells that had been exposed to the drugs for 18 h, and expressed as percent change compared to control cells (Ctrl) with vehicle. Results shown are the Mean±SD of at least three experiments. In panel B, the percentage of Annexin V-positive cells was measured in cultures of Ba/F3-EPOR VF cells exposed to varying amount of the drugs for 48 h; percentage was calculated after subtraction of background apoptosis in cells incubated without drug. Results shown are the Mean±SD of at least three experiments. (TIF) [file pone.0054826.s002.tif]

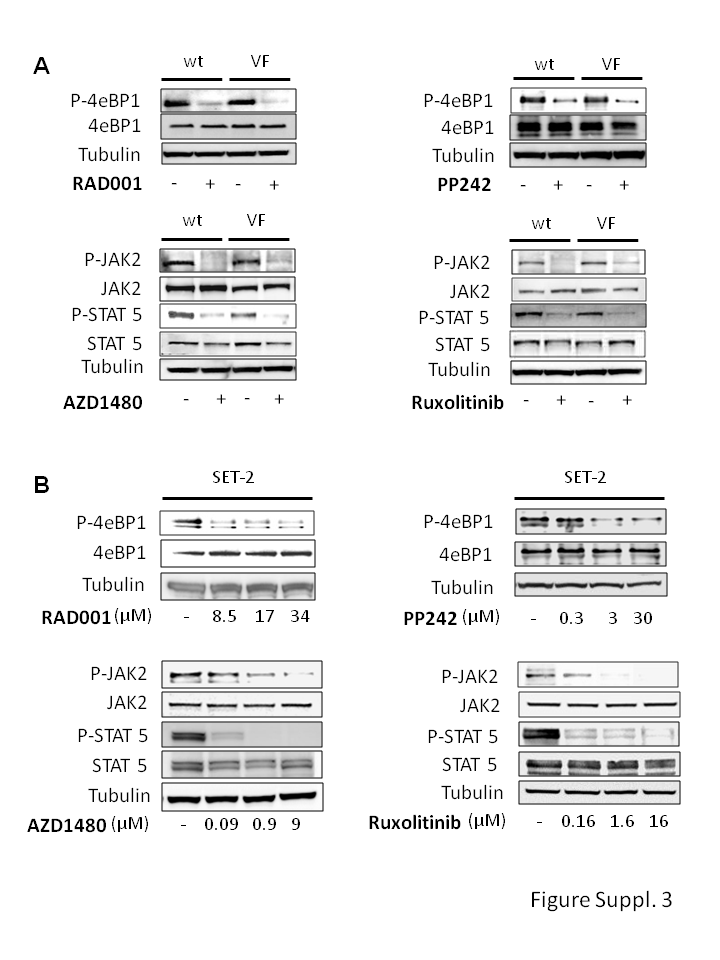

Supplement: Figure S3 — Effect of mTOR (RAD001 and PP242) and JAK2 (AZD1480 and Ruxolitinib) inhibitors on key targets in Ba/F3-EPOR VF and SET-2 cells. A. JAK2 wt and VF Ba/F3-EPOR cells were incubated for 6 h with final concentrations of the drugs corresponding to the IC50 values measured in proliferation assay (see Results for details). B. SET-2 cells were incubated for 24 h with increasing concentrations of the drugs, as indicated. The level of total and phosphorylated JAK2, STAT5, and 4EBP1 was analyzed by western blot. Tubulin was used for loading normalization. One representative of two to four similar experiments. (TIF) [file pone.0054826.s003.tif]

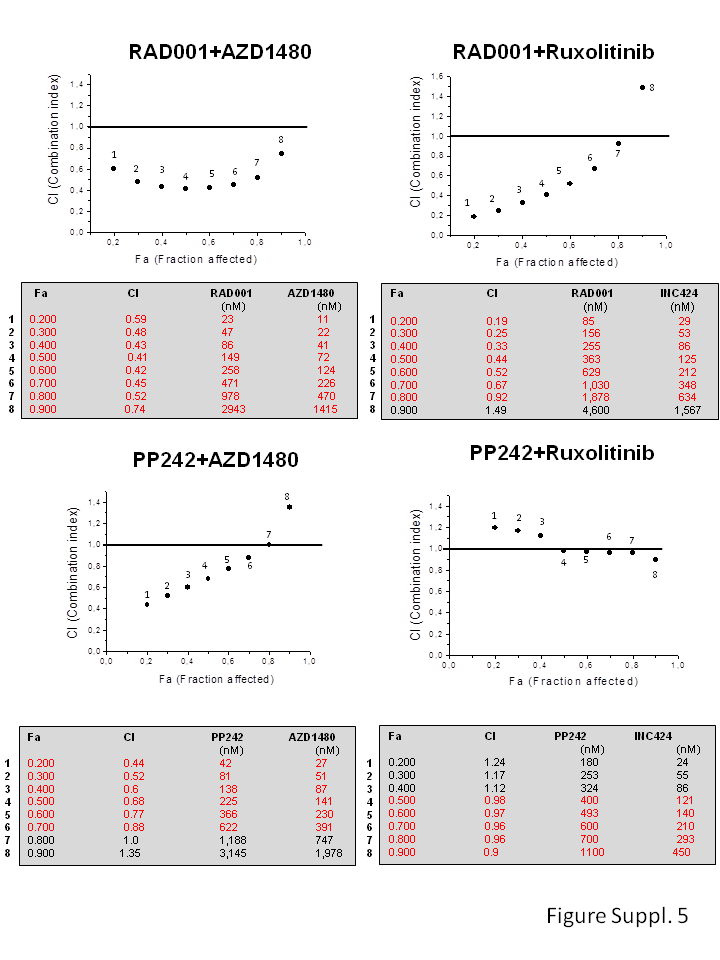

Supplement: Figure S5 — Analysis of the combinatory effects of mTOR (RAD001 and PP242) and JAK2 (AZD1480 and Ruxolitinib) inhibitors in Ba/F3-EPOR VF cells. Cells were evaluated in proliferation assay for 48 h using WST-1 assay to measure the proportion of cells alive, after subtraction of the value measured in control wells with vehicle only. “Fa” is the cell fraction affected by the dose of the drug; the combination Index (CI) was calculated as described in Materials and Methods using Calcusyn Software. A CI<1 indicates that the interaction of the two drugs is synergistic. (TIF) [file pone.0054826.s005.tif]

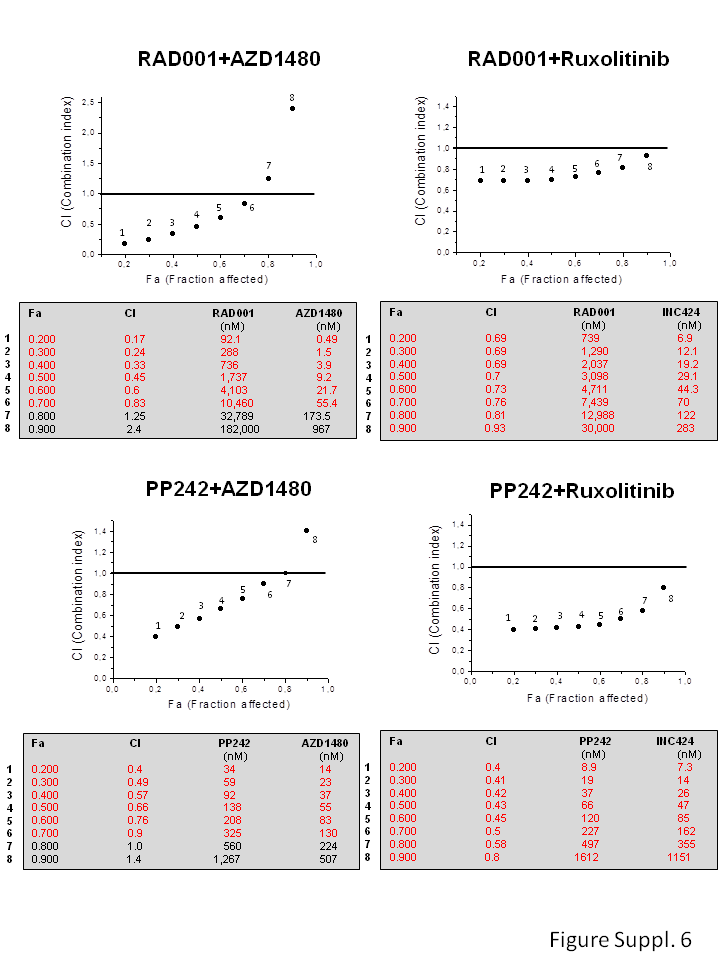

Supplement: Figure S6 — Analysis of the combinatory effects of the mTOR (RAD001 and PP242) and JAK2 (AZD1480 and Ruxolitinib) inhibitors in SET-2 cells. Cells were evaluated in proliferation assay for 48 h using WST-1 assay to measure the proportion of cells alive, after subtraction of the value measured in control wells with vehicle only. “Fa” is the cell fraction affected by the dose of the drug; the combination Index (CI) was calculated as described in Materials and Methods using Calcusyn Software. A CI<1 indicates that the interaction of the two drugs is synergistic. (TIF) [file pone.0054826.s006.tif]
